# Multi-objective genetic algorithm for synchrotron radiation beamline optimization

**DOI:** 10.1107/S1600577522010050

**Published:** 2023-01-01

**Authors:** Junyu Zhang, Pengyuan Qi, Jike Wang

**Affiliations:** a The Institute for Advanced Studies, Wuhan University, Wuhan 430072, People’s Republic of China; Uppsala University, Sweden

**Keywords:** beamline design, multi-objective optimization, genetic algorithm

## Abstract

In beamline design, there are many floating parameters that need to be tuned; manual optimization is time-consuming and laborious work, and it is also difficult to obtain well optimized results. Moreover, there are always several objectives that need to be considered and optimized at the same time, making the problem more complicated. For example, asking for both the flux and energy to be as large as possible is a usual requirement, but the changing trends of these two variables are often contradictory. In this study, a novel optimization method based on a multi-objective genetic algorithm is introduced, the first attempt to optimize a beamline with multiple objectives. In order to verify this method, beamline ID17 of the European Synchrotron Radiation Facility (ESRF) is taken as an example for simulation, with energy and dose rate as objectives. The result shows that this method can be effective for beamline optimization, and an optimal solution set can be obtained within 30 generations. For the solutions whose objectives are both improved compared with those of ESRF beamline ID17, the maximums of energy and dose rate increase by around 7% and 20%, respectively.

## Abstract 2

A universal optimization simulation method based on a multi-objective genetic algorithm is introduced; this is the first attempt to optimize the elements of a beamline using this method.

## 1. Introduction

Synchrotron radiation X-rays (Lewis, 1997 ▸; Balerna & Mobilio, 2015 ▸) have the advantages of high flux, high brilliance, high collimation and a wide energy spectrum (Winick & Doniach, 1980 ▸). Compared with the spectrum of common X-rays currently used in clinical practice, synchrotron radiation has a broad and continuous energy spectrum with tens of thousands of times higher flux, which could be of great benefit to some areas of medicine. Leveraging synchrotron radiation facilities on medical applications (computed tomography or radiotherapy) has been extensively proposed by researchers all over the world (Chicilo *et al.*, 2020 ▸; Livingstone *et al.*, 2018 ▸; Cornelius *et al.*, 2014 ▸).

Generally, the energy and dose rate of the photon beam has an important influence on the effects of treatment in radiation therapy. On one hand, the beam energy determines the position of the depth of treatment, with higher photon energy beams reaching deeper tumors (Gazda & Coia, 2001 ▸). On the other hand, radiation damage to normal tissue is less severe than that to tumors when irradiated by beams with high dose rates (normally more than 40 Gy s^−1^), which is known as the Flash effect (Durante *et al.*, 2017 ▸; Montay-Gruel *et al.*, 2018 ▸). Therefore, for beamline designers aiming at radiotherapy applications, the problem lies in how to design and arrange the beamline elements to make these two objectives, *i.e.* energy and dose rate, as large as possible.

In general, it is difficult to design beamline element parameters in a manual way, which often involves heavy hand-crafted engineering and requires beamline operators to have extensive experience. Moreover, trial-and-error procedures would also introduce individual preference or expectation, which usually makes the final solutions not optimal but empirical ones. Therefore, how to find the elements that meet the experimental requirements quickly and automatically, and obtain a photon beam with highest quality, is a problem worth discussing.

Genetic algorithms (Holland, 1992 ▸) are global optimization algorithms that can simultaneously deal with multiple individuals in a population, reducing the risk of falling into a local optimal solution, which may effectively optimize the element parameters of the beamline. Single-objective optimization problems, such as only optimizing the beam flux, have been solved successfully by using genetic algorithms (Xi *et al.*, 2015 ▸). When facing multi-objective optimization problems, a non-dominated sorting method used to select optimal solutions can be introduced. *Geant4* is a Monte Carlo simulation software which can describe well the passage of a photon beam from the source to the target (Agostinelli *et al.*, 2003 ▸). Therefore, a genetic algorithm has been applied to ESRF beamline ID17, built using *Geant4*, for simulation in order to explore the feasibility of this approach.

This paper is organized as follows. Related methods including the genetic algorithm, non-dominated sorting, *Geant4* and *SHADOW* are introduced in Section 2. The whole optimization process is introduced in Section 3, while Section 4 shows the results of the simulation. Finally, a discussion and conclusion are given in Section 5.

## 2. Methodology

### 2.1. Genetic algorithms

**Figure 1:**
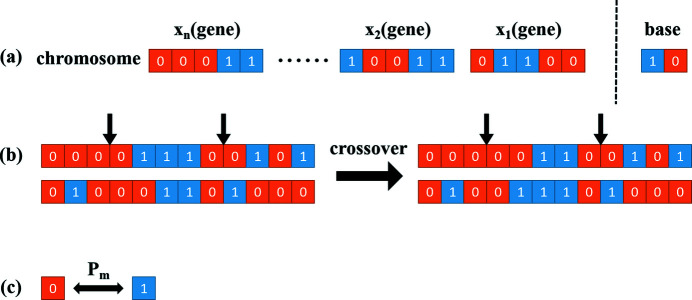
Binary encoding and genetic operators. (*a*) Parameters are converted to binary code. (*b*) The crossover operator. (*c*) The mutation operator with probability *P*_m_.

Genetic algorithms, stochastic search algorithms drawing on natural selection and natural genetic mechanisms in the biological community, were first proposed by John H. Holland in 1975 (Holland, 1975 ▸). Genetic algorithms simulate the reproduction, crossover and genetic mutation in natural selection and the natural genetic process. They obtain a set of candidate solutions in each generation. Superior individuals from the solutions are selected according to some index to combine into a new generation of candidate solutions by using some genetic operators such as selection, crossover and mutation. In general, each generation has the same number of individuals. Genetic algorithms have a number of encoding rules (Kumar, 2013 ▸), and the binary encoding rule is one of the most popular. As shown in Fig. 1 ▸(*a*), the value of each parameter is converted to binary code, and treated as a gene. In addition, each bit of binary code is considered as a base. All the genes make up one chromosome that represents an individual.

There are three genetic operators of an evolution cycle including selection, crossover and mutation. The selection operator is applied to the population, of which the purpose is to pass optimized individuals directly or to pass new individuals through crossover and mutation to the next generation. Selection is based on the fitness of individuals in the population. The crossover operator is where the chromosomes of two individuals exchange parts of their genes or bases with each other to form two new individuals, which is shown in Fig. 1 ▸(*b*). It is the main method of generating new individuals, which determines the global search capability of a genetic algorithm, thus playing a key role in the genetic algorithm. The mutation operator shown in Fig. 1 ▸(*c*) is where the genes or bases of the chromosome are replaced by other genes or bases in order to generate a new individual, which is an auxiliary method for forming new individuals determining the local search capability of the genetic algorithm. The mutation operator cooperating with the crossover operator can effectively complete the optimization process of the genetic algorithm.

For the whole optimization process of genetic algorithms, initialization is the first step, which means that a certain number of individuals are generated randomly. Then, according to fitness, the better individuals will be selected to generate the next population using the crossover and mutation operator. The cycle is continued until the population number reaches a preset value. Comparing the value of fitness of each individual is not feasible when there is more than one objective that should be optimized and where one objective improves while the other one deteriorates. Therefore, a method for selecting better individuals needs to be used, such as non-dominated sorting.

### 2.2. Non-dominated sorting

Non-dominated sorting (Deb *et al.*, 2002 ▸) is a method that uses the Pareto optimal concept (Hochman & Rodgers, 1969 ▸; Ngatchou *et al.*, 2005 ▸; Arnold, 2015 ▸). If the objectives need to be maximum, a multi-object optimization problem can be formulated as

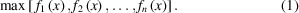

For two given feasible solutions, one of them *x*_*a*_ is said to Pareto dominate the other *x*_*b*_ if

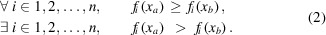

A solution *x*_*i*_ is called Pareto optimal if there is not another solution that can dominate it. The set of Pareto optimal outcomes is often called the Pareto front. In a set of solutions, the Pareto level of the Pareto optimal is defined as 1. If they are deleted from the set of solutions, the Pareto level of the Pareto optimal in the remaining solutions is defined as 2, and so on, and the Pareto level of all the solutions can be obtained.

In order to sort the solutions of the same Pareto level, crowding distance is introduced, which makes the solutions more uniform in the objective space. The crowding distance is equal to the sum of the distance between the former solution and the latter solution in the direction of each objective function.

**Figure 2:**
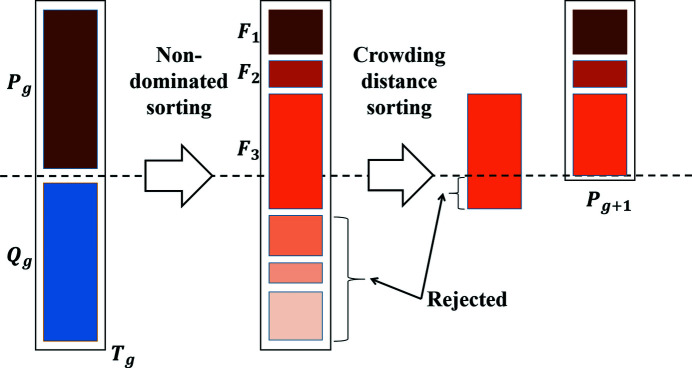
The non-dominated sorting genetic algorithm.

The specific process of non-dominated sorting is shown in Fig. 2 ▸. A parent population *P*_*g*_ generates an offspring population *Q*_*g*_ and a combined population *T*_*g*_ is obtained. The population size is more than the total number of individuals whose Pareto level is 1 or 2 but less than the total number of individuals whose Pareto level is 1 or 2 or 3. In this case, crowding distance sorting can help to reject some individuals to obtain a new parent population *P*_*g*+1_.

### 2.3. Photon transport simulation with *Geant4*

The fitness is calculated by simulating radiation transport. It is implemented through *Geant4* which is a freely available software used to perform Monte Carlo simulations of the interactions of energetic particles in matter. The reason for choosing *Geant4* is that it can calculate the energy deposition points inside the patient and collect information about the particles, as well as being flexible for users, such as being able to use Python3.7 to start the *Geant4* project.

The main function of *Geant4* is to simulate the transmission process of photons and calculate their deposited energy in the target volume. In order to avoid infrared divergence in the simulation, it is essential to set the cut range. In this study, the value of the cut range is set to 1 µm. The version of *Geant4* used was 10.2.

### 2.4. Photon generation with *SHADOW*

**Figure 3:**
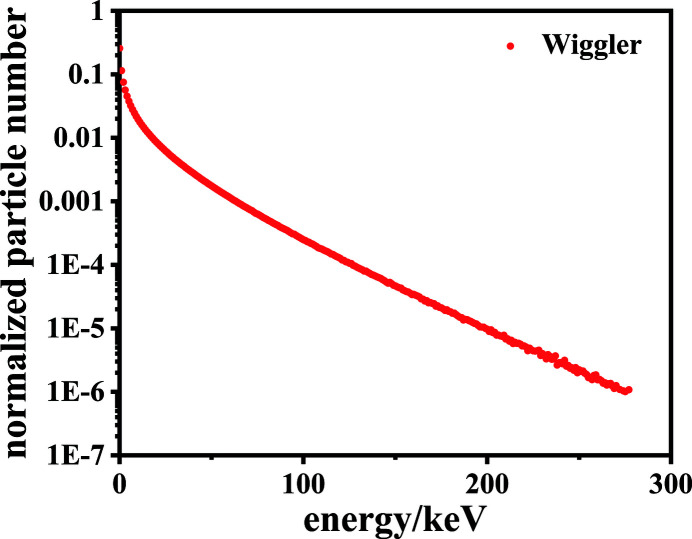
Spectrum of the photon output from the wiggler.

*Geant4* has its own way of generating a photon source, but it is difficult to simulate a synchrotron radiation source, especially to build a wiggler source that is identical to beamline ID17. In this case, *SHADOW* is used to accomplish this task. *SHADOW* (Lai *et al.*, 1988 ▸; Sanchez del Rio *et al.*, 2011 ▸) is a powerful X-ray optics ray-tracing coding developed in the early 1980s, which has been used extensively for simulations in synchrotron radiation beamline optics. Electrons emit photons in the direction of their trajectories while moving in the wiggler. Using the parameters of ID17, *SHADOW* generates a set of photons that sample the source distribution of the wiggler (the brightness function). Meanwhile, much relevant information for each photon can be obtained such as the spatial coordinates, direction, energy and so on. The parameters of ID17 (http://www.esrf.eu/) are given in Table 1 ▸ and include: electron energy, current, electron beam spot-size, horizontal and vertical divergence; and magnetic field period, number of periods, maximum magnetic field and deflection parameter *K* of the wiggler. The spectrum of the photon output from the wiggler is shown in Fig. 3 ▸.

## 3. Optimization process

The aim of beamline optimization is to maximize the energy and dose rate of the photon beam. The objective functions are

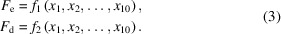

The constraints are

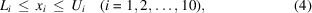

where *x*_*i*_ represents the parameter value of the beamline element, and *L*_*i*_ and *U*_*i*_ represent the lower and upper limit of the search space, respectively. The value of *x*_*i*_ of the initial generation population is generated randomly; then the offspring populations are generated by a genetic algorithm code written in Python. The values of the fitness, *F*_e_ and *F*_d_, are calculated through simulation after the value of *x*_*i*_ is put into *Geant4*.

**Figure 4:**
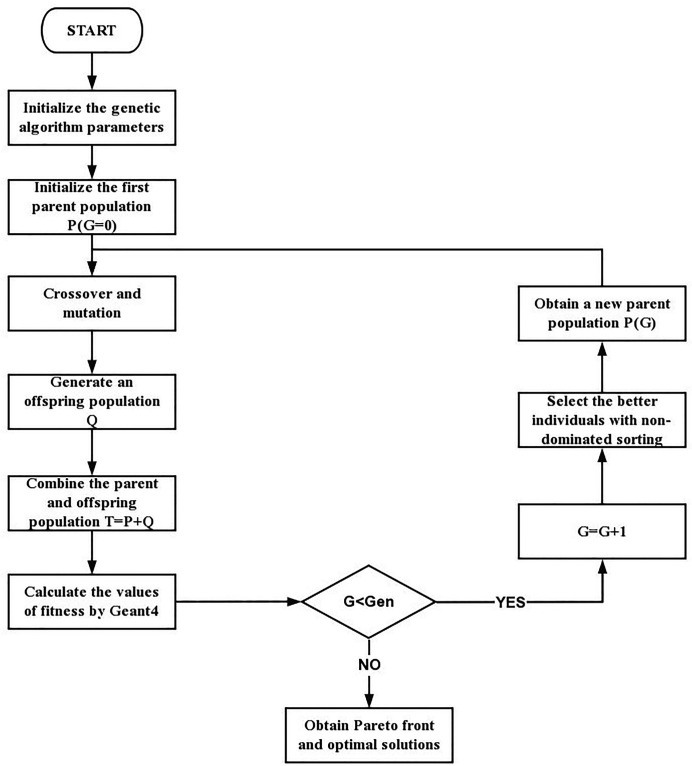
Flow chart of the genetic algorithm.

The beamline optimization of the whole process is given in Fig. 4 ▸:

(1) Initialize the genetic algorithm parameters, including the search space of *x*_*i*_, the maximum of population number (Gen), population size (*N*), crossover probability (*P*_c_) and mutation probability (*P*_m_).

(2) Repeat the process for which an individual is represented by a certain length binary code generated randomly, to form the first parent population until the number of individuals reaches the population size.

(3) Generate an offspring population from the parent population through crossover and mutation.

(4) Combine the parent and offspring population and calculate the values of fitness of each individual using *Geant4*.

(5) Evaluate the fitness for individuals in the combined population. Use a non-dominated sorting method to sort the individuals and give each an individual Pareto level.

(6) Check the end of the process. It will be terminated when the population number reaches the maximum number of iterations set at the beginning of the process. Then output the Pareto front and optimal solutions whose Pareto level is 1. Otherwise, according to the Pareto level and sorting of individuals in the combined population, a new parent population is formed and iterations continue.

## 4. Results of beamline optimization

**Figure 5:**
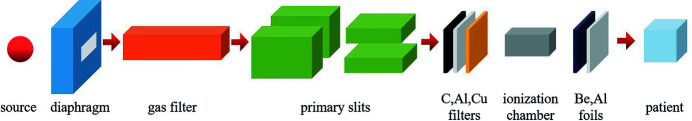
Beamline elements for the optimization simulation.

To verity the feasibility of beamline optimization using a genetic algorithm, the structure of ESRF beamline ID17 was chosen to test the simulation optimization; the layout of beamline ID17 can be seen in Fig. 5 ▸.

All the elements, including diaphragm, gas filter (Requardt *et al.*, 2013 ▸), primary slits, solid filters, ionization chamber, Be and Al foils, are constructed using *Geant4*. Meanwhile, diaphragm, primary slits and patient made of water, which can determine the cross-section size of the photon beam, are fixed at 21.6 m, 29.3 m and 40.5 m, respectively. The other elements influence the optimization objectives – energy and dose rate. Besides the position of the gas filter, solid filters, ionization chamber and Be and Al foils, there are some other parameters to be optimized, such as the length of the gas filter, the type and pressure of the gas inside, and the thickness of C, Al and Cu. All parameters and their ranges are shown in Table 2 ▸.

In this study, a genetic algorithm code is run using Python with the parameters *N* = 180, Gen = 200, *P*_c_ = 0.8 and *P*_m_ = 0.2, in order to find a reasonable optimal solution with the best combination of beamline elements, and to verify the effectiveness of the whole optimization process. After testing, these parameters were found to be suitable for the simulation results.

**Figure 6:**
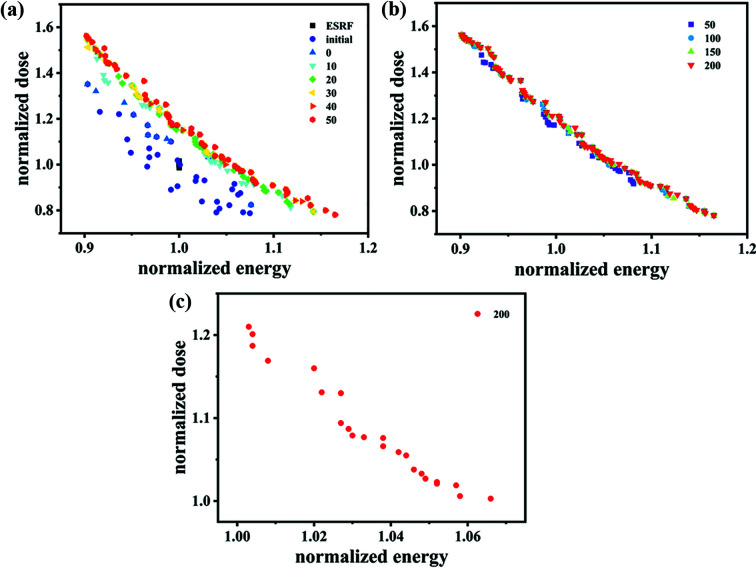
The results of the optimization simulation. (*a*) The solutions for ESRF are represented by the black squares and the initial populations are represented by purple circles as well as the Pareto front of the population with the generation number of 0, 10, 20, 30, 40 and 50. (*b*) The Pareto front of the population with the generation number of 50, 100, 150, 200. (*c*) The solutions of the last generation that are better than that of the ESRF.

In the optimization model, two objectives are considered – maximizing the energy and maximizing the dose rate simultaneously. Thus, making use of non-dominated sorting to find the Pareto front is applied to the model. The Pareto fronts of some generations are given in Fig. 6 ▸. The *X*-coordinate is named the normalized energy, because the energy of each individual is divided by the average energy of ESRF beamline ID17 through a five times simulation, and similarly for the *Y*-coordinate. Although the output is the dose rather than the dose rate, the same light source and the same number of photons make the dose rate equivalent to the dose.

A convergence check is used to judge whether the genetic algorithm is terminated. In Fig. 6 ▸(*a*), the solutions of the initial generation are randomly generated so the distribution is homogeneous. With the generation number growing, the solutions in the Pareto front gradually move in the direction of high energy and high dose. As can be seen in Figs. 6 ▸(*a*) and 6 ▸(*b*), the Pareto front achieves a stable convergence at a generation whose number is approximately between 20 and 30. It does not change much up to the generation of 200.

The solutions that are better than that of the ESRF are selected from the optimal solutions of the last generation, which are located at the upper right of the black squares in Fig. 6 ▸(*a*). In other words, the solutions whose normalized energy and normalized dose are both greater than 1 will be satisfactory. As shown in Fig. 6 ▸(*c*), there are 23 optimal solutions obtained, among which the maximum of energy reaches 1.066, a 6.6% improvement relative to ESRF, and the maximum of dose reaches 1.21, a 21% improvement relative to that of the ESRF. Besides, other optimal solutions in the Pareto front which are not shown in Fig. 6 ▸(*c*) also have varying degrees of optimization in a single aspect of energy or dose. This means that, although their dose (or energy) is lower, their energy (or dose) is higher than that of the ESRF.

## 5. Discussion and conclusion

A genetic algorithm is a global optimization algorithm that can simultaneously deal with multiple individuals in a population, reducing the risk of falling into a local optimal solution. When the problem involves multi-objective optimization, there are still ways to use a genetic algorithm, such as by non-dominated sorting. In this work, a simulation for beamline optimization based on a genetic algorithm is accomplished to verify the effectiveness and feasibility of the genetic algorithm. The results show that the Pareto front can achieve a stable convergence at a generation whose number is approximately between 20 and 30. There are 23 optimal solutions better than that of the ESRF for a generation number of 200, among which the maximum of energy increases by 6.6% and the maximum of dose increases by 21%. Many optimal solutions can be obtained through this multi-objective genetic algorithm. In the absence of more information, it is impossible to determine which solution is better. In general, various related constraints are added to the genetic algorithm according to the requirements of the experiment and beamline design. Then the appropriate solution is selected after a set of optimal solutions is obtained. For example, this method can be used to design Flash therapy beamlines, since Flash therapy requires a high dose rate and as much energy as possible for the treatment. Moreover, other kinds of beamline besides medical beamlines can also be optimized by this method due to the characteristics of the genetic algorithm.

## Figures and Tables

**Table 1:** ESRF electron beam parameters and ID17 wiggler parameters

Electron beam parameters			Wiggler parameters	
Electron energy	6.04 GeV		Magnetic field period	15 cm
Current	0.2 A		Number of periods	11
Horizontal beam size	0.057 mm		Maximum magnetic field	1.592 T
Vertical beam size	0.0103 mm		Deflection parameter *K*	22.30
Horizontal divergence	3.9 nm			
Vertical divergence	0.039 nm			

**Table 2:** Parameters needed to be optimized and their ranges

Length of gas filter	0.50–2.50 m
Position of gas filter	23.40–27.65 m
Pressure of gas	0.030–0.300 bar
Type of gas	Ar, Kr, Xe
Thickness of C	0.10–4.00 mm
Thickness of Al	0.10–4.00 mm
Thickness of Cu	0.10–4.00 mm
Position of solid filters	29.70–32.00 m
Position of ionization chamber	32.10–36.00 m
Position of Be and Al foils	36.20–40.20 m

## References

[bb1] Agostinelli, S., Allison, J., Amako, K., Apostolakis, J., Araujo, H., Arce, P., Asai, M., Axen, D., Banerjee, S., Barrand, G., Behner, F., Bellagamba, L., Boudreau, J., Broglia, L., Brunengo, A., Burkhardt, H., Chauvie, S., Chuma, J., Chytracek, R., Cooperman, G., Cosmo, G., Degtyarenko, P., Dell’Acqua, A., Depaola, G., Dietrich, D., Enami, R., Feliciello, A., Ferguson, C., Fesefeldt, H., Folger, G., Foppiano, F., Forti, A., Garelli, S., Giani, S., Giannitrapani, R., Gibin, D., Gómez Cadenas, J. J., González, I., Gracia Abril, G., Greeniaus, G., Greiner, W., Grichine, V., Grossheim, A., Guatelli, S., Gumplinger, P., Hamatsu, R., Hashimoto, K., Hasui, H., Heikkinen, A., Howard, A., Ivanchenko, V., Johnson, A., Jones, F. W., Kallenbach, J., Kanaya, N., Kawabata, M., Kawabata, Y., Kawaguti, M., Kelner, S., Kent, P., Kimura, A., Kodama, T., Kokoulin, R., Kossov, M., Kurashige, H., Lamanna, E., Lampén, T., Lara, V., Lefebure, V., Lei, F., Liendl, M., Lockman, W., Longo, F., Magni, S., Maire, M., Medernach, E., Minamimoto, K., Mora de Freitas, P., Morita, Y., Murakami, K., Nagamatu, M., Nartallo, R., Nieminen, P., Nishimura, T., Ohtsubo, K., Okamura, M., O’Neale, S., Oohata, Y., Paech, K., Perl, J., Pfeiffer, A., Pia, M. G., Ranjard, F., Rybin, A., Sadilov, S., Di Salvo, E., Santin, G., Sasaki, T., Savvas, N., Sawada, Y., Scherer, S., Sei, S., Sirotenko, V., Smith, D., Starkov, N., Stoecker, H., Sulkimo, J., Takahata, M., Tanaka, S., Tcherniaev, E., Safai Tehrani, E., Tropeano, M., Truscott, P., Uno, H., Urban, L., Urban, P., Verderi, M., Walkden, A., Wander, W., Weber, H., Wellisch, J. P., Wenaus, T., Williams, D. C., Wright, D., Yamada, T., Yoshida, H. & Zschiesche, D. (2003). *Nucl. Instrum. Methods Phys. Res. A*, **506**, 250–303.

[bb2] Arnold, B. C. (2015). *Wiley Statsref: Statistics Reference Online*, pp. 1–10. Wiley.

[bb3] Balerna, A. & Mobilio, S. (2015). In *Synchrotron Radiation: Basics, Methods and Applications*, edited by S. Mobilio, F. Boscherini and C. Meneghini, pp. 3–28. Berlin, Heidelberg: Springer-Verlag.

[bb4] Chicilo, F., Hanson, A., Geisler, F., Belev, G., Edgar, A., Ramaswami, K., Chapman, D. & Kasap, S. (2020). *Phys. Med. Biol.* **65**, 075010. 10.1088/1361-6560/ab7361 32242527

[bb5] Cornelius, I., Guatelli, S., Fournier, P., Crosbie, J. C., Sanchez del Rio, M., Bräuer-Krisch, E., Rosenfeld, A. & Lerch, M. (2014). *J. Synchrotron Rad.* **21**, 518–528. 10.1107/S1600577514004640 24763641

[bb6] Deb, K., Pratap, A., Agarwal, S. & Meyarivan, T. (2002). *IEEE Trans. Evol. Comput.* **6**, 182–197.

[bb7] Durante, M., Bräuer-Krisch, E. & Hill, M. (2017). *Br. J. Radiol.* **91**, 20170628. 10.1259/bjr.20170628 PMC5965780 29172684

[bb9] Gazda, M. J. & Coia, L. R. (2001). In *Cancer Management: A Multidisciplinary Approach: Medical, Surgical and Radiation Oncology*, edited by R. Pazdur, L. R. Coia, W. J. Hosians and L. D. Wagman, pp. 9–19. Melville: PRR Inc.

[bb10] Hochman, H. M. & Rodgers, J. D. (1969). *Am. Econ. Rev.* **59**, 542–557.

[bb922] Holland, J. H. (1975). *Adaptation in Natural and Artificial Systems.* University of Michigan Press.

[bb11] Holland, J. H. (1992). *Sci. Am.* **267**, 66–72.

[bb12] Kumar, A. (2013). *Int. J. Adv. Res. IT. Eng.* **2**, 1–7.

[bb13] Lai, B., Chapman, K. & Cerrina, F. (1988). *Nucl. Instrum. Methods Phys. Res. A*, **266**, 544–549.

[bb14] Lewis, R. (1997). *Phys. Med. Biol.* **42**, 1213–1243. 10.1088/0031-9155/42/7/001 9253036

[bb15] Livingstone, J., Stevenson, A., Häusermann, D. & Adam, J. (2018). *Phys. Med.* **45**, 156–161. 10.1016/j.ejmp.2017.12.017 29472081

[bb16] Montay-Gruel, P., Bouchet, A., Jaccard, M., Patin, D., Serduc, R., Aim, W., Petersson, K., Petit, B., Bailat, C., Bourhis, J., Bräuer-Krisch, E. & Vozenin, M. C. (2018). *Radiother. Oncol.* **129**, 582–588. 10.1016/j.radonc.2018.08.016 30177374

[bb17] Ngatchou, P., Zarei, A. & El-Sharkawi, A. (2005). *Proceedings of the 13th International Conference on Intelligent Systems Application to Power Systems (ISAP2005)*, 6–10 November 2005, Arlington, VA, USA, pp. 84–91.

[bb18] Requardt, H., Renier, M., Brochard, T., Bräuer-Krisch, E., Bravin, A. & Suortti, P. (2013). *J. Phys. Conf. Ser.* **425**, 022002.

[bb19] Sanchez del Rio, M., Canestrari, N., Jiang, F. & Cerrina, F. (2011). *J. Synchrotron Rad.* **18**, 708–716. 10.1107/S0909049511026306 PMC3267628 21862849

[bb20] Winick, H. & Doniach, S. (1980). *Synchrotron Radiation Research*, pp. 11–25 New York: Plenum.

[bb21] Xi, S., Borgna, L. S. & Du, Y. (2015). *J. Synchrotron Rad.* **22**, 661–665. 10.1107/S1600577515001861 25931082

